# Nurse-Initiated Fall and Osteoporosis Screening for Older Adults in the Emergency Department

**DOI:** 10.7759/cureus.36001

**Published:** 2023-03-10

**Authors:** Yi-En C Seah, Shu Fang Ho, Arron Seng Hock Ang, Jayvilyn P Bacud, Barbara H Rosario

**Affiliations:** 1 Emergency Medicine, Changi General Hospital, Singapore, SGP; 2 Emergency Medicine, Singapore General Hospital, Singapore, SGP; 3 Geriatric Medicine, Changi General Hospital, Singapore, SGP

**Keywords:** osteoporosis, adult emergency department, fall assessment, elderly falls, frailty screening

## Abstract

Background

Many older adults presenting to the emergency department (ED) after a fall are discharged without adequate assessment of their fall risk. A nurse-initiated protocol was introduced for the early screening of older adults with injurious falls. We aimed to promote osteoporosis education and right-site them to appropriate outpatient resources in the community.

Methodology

In this study, we included ≥65-year-old adults who attended the ED with injurious falls or near falls between December 2019 and December 2020. An ED nurse trained in basic geriatric care performed the cognitive assessment and provided advice on diet, footwear, fall safety, calcium/vitamin D supplementation, and osteoporosis screening.

Results

A total of 70 (75.7% female) patients aged 65-93 years were included. In total, 34 (48.6%) were started on calcium/vitamin D supplements and 22 (31.4%) went on to receive outpatient bone mineral density scans. Only three patients reattended the ED for recurrent falls/fractures in the six-month follow-up period.

Conclusions

A nurse-initiated fall and osteoporosis screening protocol is a feasible model of care for targeted screening and education of older adults who present to the ED with injurious falls.

## Introduction

Older adults and falls

Falls are the second leading cause of unintentional injury deaths worldwide. Older people have the highest risk of death or serious injury arising from a fall and the risk increases with age [1]. The population of older adults aged 65 years and above in Singapore has increased from 9% in 2010 to 16% in 2021 [2]. By 2035, it is estimated that around one-third of Singaporeans will be aged 65 and above, while the median age is also expected to rise from 39.7 in 2015 to 53.4 in 2050 [3]. In Singapore, the incidence rate of unintentional falls in 2012 was 278 per 100,000 for adults aged 60 years and older [4]. Patients who fall are at risk of recurrent falls, and 30% will fall again if their risk factors are not properly addressed [5]. In addition, a rapidly aging population is at higher risk of age-related disabilities, such as poor mobility, visual impairment, hearing loss [6], cognitive impairment, osteoporosis, and frailty, some of which may not become apparent until the impairment is advanced but treatable if identified with early screening [7]. Therefore, timely referrals for appropriate follow-up including osteoporosis screening and treatment can reduce the risk of falls and injurious outcomes [5].

Osteoporosis prevalence and costs in Singapore

Singapore is currently facing an increasing burden of osteoporosis and osteoporosis-related fractures. In 2009, the International Osteoporosis Foundation (IOF) Asian Audit reported that 55,000 women above 50 years of age in Singapore suffer from osteoporosis, and this number is likely to increase significantly with an aging population [8]. Osteoporosis is often only diagnosed after a fracture has occurred, and treatment is delayed or not started in older adults with fractures locally and worldwide [9-11].

The clinical and public health implications of osteoporosis are largely attributed to fractures due to the disability suffered and loss of healthy life years as a result. The incidence of osteoporotic fractures is projected to increase, and the total economic burden (including direct costs and indirect costs to society) associated with these fractures was estimated at S$183.5 million in 2017 and is forecasted to reach S$289.6 million by 2035. Increasing the treatment rate for osteoporosis can avert up to 29,096 fractures over the forecast period (2017-2035), generating cumulative total cost savings of up to S$330.6 million [12].

Need for a fracture liaison program

A local retrospective study highlighted a statistically significant treatment gap in the diagnosis and treatment of osteoporosis in fragility fracture patients in a regional hospital as well as the need for a standardized osteoporosis liaison program in Singapore’s regional health systems [11]. The Asia Pacific Consortium on Osteoporosis (APCO) Framework advises clinicians on the clinical standards of care for the screening, diagnosis, and management of osteoporosis in the Asia-Pacific region. Some of the recommendations include having a systematic and proactive bone health assessment for patients who sustain a fragility fracture (Clinical standard 1), use of country-specific fracture risk assessment tools if available (e.g., Fracture Risk Assessment Tool (FRAX®), Garvan, etc.) for prediction of future fracture risk and/or osteoporosis risk (Clinical standard 5), and doing a fall risk assessment as a standard component of the investigation of an individual’s future fracture risk (Clinical standard 7) [13].

Likewise, a meta-analysis showed that more intensive Fracture Liaison Service programs, which include components of screening, assessing, educating, and treating older adults with falls, and concurrent alerting of their primary care providers in the period immediately following a fragility fracture, are associated with increased bone mineral density (BMD) assessment and osteoporosis treatment initiation [14].

Aims and objectives

Many older adults (≥65 years old) who attend the emergency department (ED) following a fall resulting in a fracture will be referred to the orthopedics fracture clinic without an in-depth fall assessment for future fall risk. The aim of the plaster room protocol is to provide a low-cost, minimal-risk opportunity to optimize the care of older adults with injurious falls being discharged home from the ED.

This paper describes the introduction of a nurse-initiated plaster room protocol for early fall screening and osteoporosis education of older adults with injurious falls or near falls. It is undertaken during the time the patient receives their plaster cast in the ED before discharge home. This is in line with the American Geriatrics Society fall prevention guidelines which recommend a multifactorial fall risk assessment for all older adults who present with a fall or who have gait and balance problems and includes interventions such as foot and footwear advice, vitamin D supplementation in all older adults, and education of patients and caregivers [15].

## Materials and methods

Study design, setting, and stakeholders

**Figure 1:**
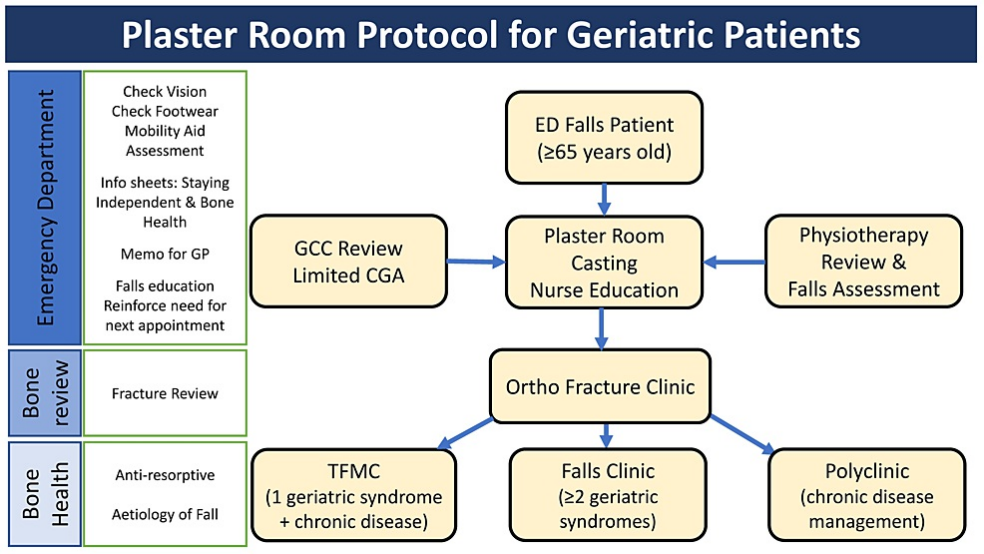
Emergency department plaster room protocol. GP: general practitioner; ED: emergency department; GCC: Geriatric Care Champion; CGA: comprehensive geriatric assessment; Ortho: orthopedics; TFMC: Tampines Family Medicine Clinic

The plaster room protocol (Figure 1) was introduced in the ED of a 1,000-bed public hospital in Singapore in December 2019 after approval from the hospital’s Medical Board. Patients aged 65 years or older attending the ED for falls or near falls during office hours (Monday to Friday, except public holidays, between 8 am and 4 pm) were screened. Initially, we only included patients with upper limb (UL) fractures, but after successful implementation, it was expanded to include all older adults with injurious falls or near falls planned for discharge home from the ED. It is nurse-initiated with geriatrician support at the initial implementation of the protocol.

The protocol involves opportunistic screening by a Geriatric Care Champion (GCC), an ED nurse trained in geriatric care. In addition to the usual plaster cast care advice and referral to the orthopedics fracture clinic, patients also undergo the interventions listed in Table 1.

**Table 1:** Interventions by Geriatric Care Champion in the emergency department.

Interventions by Geriatric Care Champion
Targeted geriatric assessment to assess functional status, cognition, fall risk, need for mobility aids/physiotherapist assessment, and other geriatric syndromes
Education on fall prevention, bone health, and use of mobility aids (if needed)
Provide advice on initiation of bone health screening and education on dietary interventions to prevent sarcopenia such as recommending the attending clinician to prescribe calcium and vitamin D supplements, or to reinforce patient compliance if already on treatment
Right-site to the most appropriate outpatient resource (primary care vs. specialist clinic) for bone health assessment and comprehensive geriatric assessment (CGA), with advice to undergo osteoporosis screening and treatment by the primary care provider via a standardized General Practitioner memo (Appendices)

As part of a larger Geriatric Services Hub (GSH) project with the Geriatric Education and Research Institute (GERI), we partnered with a general practice clinic, the Tampines Family Medicine Clinic (TFMC) [16], aiming to provide affordable integrated care for frail older adults in the community.

Data collection and analysis

A retrospective review of the patients seen under this protocol was undertaken to assess compliance and effectiveness of the protocol. Ethics exemption was granted by the Centralized Institutional Review Board.

Electronic patient records were reviewed and data collection included demographic data for age, gender, and race. Other data included type of injury, fall history, osteoporosis history, functional assessments, discharge disposition, and six-month unplanned ED re-attendance and/or hospital admission for falls or fragility fracture. Patient compliance with follow-up and calcium supplementation were reviewed. Onward referrals were assessed for compliance with bone health recommendations, in particular, BMD screening, the continuation of calcium/vitamin D supplementation, and whether an anti-resorptive treatment was initiated. Compliance with medications was assumed if treatment was started and continued during the six-month review period. Future fracture risk was retrospectively calculated using the FRAX® for patients with available data. All data were analyzed using Microsoft Excel 2016.

## Results

Patient profile

A total of 70 patients were screened between December 2019 and December 2020. They were aged 65 to 93 years old, and the majority (75.7%) were female.

A total of 67 (95.7%) patients had fallen and three had a near fall. As a consequence, 49 (70%) sustained an UL fracture, 13 (18.6%) suffered a lower limb (LL) fracture, and one (1.4%) had a lumbar compression fracture. The rest had non-fracture injuries of the UL (five patients) and LL (two patients). Of note, 21 (30%) patients had a history of falls within 12 months of their current ED visit.

The median basic activities of daily living (bADL) score was 20 (n = 59, range = 15-20), and the median instrumental activities of daily living (iADL) score was 7 (n = 59, range = 0-7), of which higher scores would indicate a better functional status for the patient. The median abbreviated mental test (AMT) score was 7 (n = 49, range = 5-10), which assesses patients’ cognition, and a score of 7 or more would signify no significant cognitive impairment.

In total, four (5.7%) patients were known to have osteoporosis, and one patient had pre-existing osteopenia. Fifteen (21.4%) patients were already on calcium/vitamin D supplementation, of which three (4.3%) were concurrently on bisphosphonates. The median retrospectively calculated FRAX® (without BMD) for hip fracture risk was 3.9% (n = 57, range = 0.8-18%), and the overall fracture risk was high at 11% (n = 57, range = 3.3-25%). A summary of the patient profile is shown in Table 2.

**Table 2:** Patient profile. *n <70 due to missing data. FRAX®: Fracture Risk Assessment Tool; BMD: bone mineral density

Patient profile	Number (n = 70)	Percentage %
Gender		
Male	17	24.3%
Female	53	75.7%
Age group (years)		
65–70	19	27.1%
71–80	36	51.4%
>81	15	21.4%
Race		
Chinese	56	80.0%
Malay	10	14.3%
Indian	1	1.4%
Others	3	4.3%
Mechanism of injury		
Fall	67	95.7%
Near fall	3	4.3%
Injury profile		
Upper limb (fracture)	49	70.0%
Upper limb (non-fracture)	5	7.1%
Lower limb (fracture)	13	18.6%
Lower limb (non-fracture)	2	2.9%
Lumbar compression fracture	1	1.4%
Past medical history		
History of a fall in the previous 12 months	21	30.0%
Known osteopenia	1	1.4%
Known osteoporosis	4	5.7%
On calcium/vitamin D supplements	15	21.4%
On bisphosphonate	3	4.3%
Functional scores	n^*^	Range (median)
Basic activities of daily living	59	15–20 (20)
Instrumental activities of daily living	59	0–7 (7)
Abbreviated Mental Test (AMT)	49	5–10 (7)
Retrospective FRAX^®^ fracture risk	n^*^	Range (median)
Overall fracture risk (without BMD)	57	3.3–25% (11%)
Hip fracture risk (without BMD)	57	0.8–18% (3.9%)

Intervention outcomes

The majority of the patients (94.3%) were referred to Orthopedics Specialist Outpatient Clinic (SOC) for follow-up of their fracture and other appointments depending on their individual clinical issues, focusing on their need for additional bone health assessment and CGA.

A total of 22 (31.4%) patients received a BMD screen through outpatient services. Among the 70 patients screened, 34 (48.6%) were prescribed calcium/vitamin D on discharge from the ED. The reasons for non-prescription included the patient’s refusal or the patient having his/her own supply (15 patients were already on calcium supplementation before presentation). In total, 39 (55.7%) patients were noted to have continued their supplements at the six-month follow-up, with compliance as high as 80% (12 out of 15 patients) among those who were already on supplements prior to the index ED visit, and 70.6% (24 out of 34 patients) among those newly started from the ED. The most common reason for discontinuation was that the patient defaulted on their follow-up appointment(s). Eight (11.4%) patients were started on bisphosphonate and one (1.4%) on teriparatide after a review of their bone health.

The ED re-attendance for falls/fractures at six months after the index presentation at ED was low with three patients (4.3%). One patient had a recurrent fall within one week and two patients within six months, with one of the above patients sustaining a neck of femur fracture. There was no mortality at the six-month follow-up. A summary of the intervention outcomes is presented in Table 3.

**Table 3:** Intervention outcomes from the plaster room protocol. *Includes patients already on calcium/vitamin D supplements prior to the index ED visit. TFMC: Tampines Family Medicine Clinic; ED: emergency department; BMD: bone mineral density

Intervention outcomes	Number (n = 70)	Percentage %
Disposition on discharge		
Admitted to the inpatient ward	3	4.3%
Ophthalmology referral	4	5.7%
Otorhinolaryngology referral	2	2.9%
Endocrine referral	1	1.4%
Geriatrics referral	12	17.1%
Orthopedics referral	66	94.3%
Primary care - Polyclinic referral	17	24.3%
Primary care - TFMC referral	13	18.6%
Osteoporosis intervention		
Calcium/Vitamin D newly initiated by the ED	34	48.6%
Calcium/Vitamin D continued at the six-month follow-up^*^	39	55.7%
BMD screen by outpatient (total)	22	31.4%
BMD screen at Primary care - polyclinic	3	4.3%
BMD screen at Primary care - TFMC	4	5.7%
BMD screen at Specialist Outpatient Clinic	15	21.4%
Bisphosphonates initiated outpatient (total)	8	11.4%
Bisphosphonates initiated at primary care - polyclinic	2	2.9%
Bisphosphonates initiated at primary care - TFMC	1	1.4%
Bisphosphonates initiated at Specialist Outpatient Clinic	5	7.1%
Teriparatide initiated (inpatient ward)	1	1.4%
ED re-attendance for falls/fractures		
Within one week	1	1.4%
Within six months	2	2.9%
Mortality	0	0.0%

## Discussion

Calcium/vitamin D supplements and fracture risk

We note that there were only 15 (21.4%) patients on calcium/vitamin D supplements prior to their ED attendance, while the median retrospectively calculated FRAX® (without BMD) for hip fracture risk was 3.9% and the overall fracture risk was high at 11%. The FRAX® was developed to estimate the 10-year fracture risk of both hip fracture and the overall risk of a major osteoporotic fracture (clinical spine, forearm, hip, or shoulder fracture). It is based on individual patient models that integrate risks associated with clinical risk factors and BMD at the femoral neck if available [17]. While there is no internationally standardized cut-off for which osteoporosis treatment should be initiated, factors taken into consideration include age, BMD, and risk factors for fractures, falls, or bone loss. Locally, a hip fracture risk above 3% would trigger the initiation of osteoporosis treatment.

Calcium/Vitamin D can be used for the prevention and treatment of osteoporosis. There is evidence that calcium supplements have significant beneficial effects on bone density in postmenopausal women [18]. Yet, many Singaporeans are not consuming enough calcium [19], and supplements may be necessary for some. In a meta-analysis with more than 52,000 patients, a significant reduction of fragility hip fracture risk was noted for patients on calcium/vitamin D (risk ratio (RR) = 0.87) but not with calcium alone [20]. Calcium/Vitamin D supplements are easily obtainable and low-cost. In our ED, it costs just S$1.50 for 60 tablets of calcium carbonate 450 mg/Vitamin D 200 units (two tablets per day for 30 days). We postulate that a reason for medication non-compliance might be due to polypharmacy associated with multi-morbidity which tends to affect older adults [21].

Bone mineral density screening

Even though the future fracture risk is increased following a fragility fracture, a systematic review has shown that only 10-20% of patients with osteoporosis are diagnosed, and only 8% commence treatment after a fragility fracture [22]. Under our protocol, 22 (31.4%) of our recruited patients received BMD screening by outpatient services, and 39 (55.7%) patients were noted to have continued their supplements at the six-month follow-up. Likewise, similar overseas intervention programs that promote osteoporosis and bone health education after a fragility fracture have shown an increase in BMD evaluation and osteoporosis medication treatment [23,24].

Fall intervention

Falls account for a significant number of fractures in patients with osteoporosis, therefore, reducing the fall risk can help prevent fractures. When a fall is not assessed adequately, this may lead to recurrent falls, and 30% of fallers will fall again if the risk factors are not properly addressed [5]. Similarly, 30% of our recruited patients had a history of a fall in the 12-month period prior to the index presentation at ED. With the implementation of our protocol in the ED, only three (4.3%) had an unplanned ED re-attendance for a recurrent fall within six months. Likewise, a similar-sized local study showed that early geriatric specialist interventions at the ED can reduce potentially avoidable acute admissions without escalating the risk of rehospitalization, ED re-attendance, or mortality, and with possible benefit in attenuating frailty progression [25].

Opportunistic recruitment

This ED Plaster Room Protocol for geriatric patients is a nurse-initiated protocol. To our best knowledge, it is the first nurse-initiated screening protocol for older adults among all EDs in Singapore. It does not require an active referral from the ED provider in charge who can focus on the acute medical care needs of the patient. The GCC on duty identifies suitable patients via the electronic ED dashboard and opportunistically screens patients who fit the inclusion criteria. Additionally, patients can also be referred to the GCC when identified by other ED providers. This protocol was initially designed with a focus on just older fall patients with UL fractures, but due to its success, it was expanded to all other older patients with injurious falls (with or without UL fractures).

The patients who were screened were mostly independent, as evidenced by a median bADL score of 20 and an iADL score of 7 (higher scores indicate better functional status). With active screening for osteoporosis and initiating treatment early, we hope to be able to keep these patients active while decreasing their risk of osteoporosis-related complications such as fractures which can be debilitating and cause increased mortality and morbidity.

Tampines Family Medicine Clinic model for follow-up care

TFMC is a general practice clinic we partnered with as part of a larger GSH pilot program under GERI. The aim of the program is to provide affordable integrated care for frail older adults in the community [16]. There is support from our hospital’s geriatricians, and patients referred to TFMC for follow-up also benefit from governmental subsidies for select tests and medications under this scheme so that care can remain affordable and patients can remain in the community.

Of the 13 patients who were referred to TFMC, only five (38.5%) attended the appointment. Patients who defaulted were unfortunately not contactable to find out their reason for non-attendance. Potential reasons could be that they already had a regular primary care provider or they did not see the need for bone health assessment. At TFMC, four (80%) patients went on to receive a BMD scan and one started bisphosphonates. At six months, three of the five patients who attended the initial appointment at TFMC were still on active follow-up and continued on calcium supplements. This scheme potentially can be expanded to other general practice clinics in local neighborhoods to develop a decentralized model of care to ease workload constraints in acute hospitals’ SOCs and polyclinics.

Limitations

There are a few limitations with the implementation of this protocol, of which the main limitation is the lack of manpower. There is usually one GCC on duty each day, hence, the protocol is available only during office hours and covers the entire ED, including the ED short-stay observation unit. Their screening of patients via the ED dashboard is opportunistic. Therefore, some eligible patients may have been missed. Ideally, there should be an automated process where older adults presenting with injurious falls are highlighted to GCCs for assessment.

Second, only patients who were planned for discharge from the ED were included. They may not have a long window for intervention compared to those who are getting admitted to the hospital, and some may decline to stay slightly longer for counseling and education. There is potential to expand to all older adult fallers regardless of the time of presentation or disposition status if manpower allows as these patients can present to the ED at any time.

As this is a retrospective review, there are missing data (e.g., weight, height, and functional scores) that may skew the data and introduce bias into the study, as well as affect our ability to retrospectively calculate the FRAX® fracture risk for the patients.

Furthermore, re-attendance and re-admission data are only available if the patient re-attended hospitals or polyclinics using the same electronic medical system due to local personal data protection laws. Patients may have attended private healthcare providers who may have arranged bone health assessments unknown to the authors and be continued on calcium/vitamin D supplements. Therefore, our results may underrepresent those who have received bone health assessments or are still on calcium/vitamin D supplements.

## Conclusions

Many older adults do not access medical care until they face a crisis and do not participate in preventative healthcare. Hence, any contact with the healthcare system provides an opportunity for healthcare workers to intervene with education and onward referrals for necessary assessments. However, the care of older adults requires time to explore not just their medical but also functional issues. In a busy ED with an annual attendance of over 123,000, it is challenging to be able to explore all these adequately in one ED visit by the attending ED provider, and additional concurrent targeted screening by a geriatric-trained nurse can add value to their healthcare journey.

In conclusion, a nurse-initiated ED plaster room protocol is a feasible model for targeted screening of older adults who present to the ED after an injurious fall. It can allow for opportunistic fall assessment and bone health education, aiming to improve patients’ bone health and decrease future fracture risk.

## Figures and Tables

**Figure 2:**
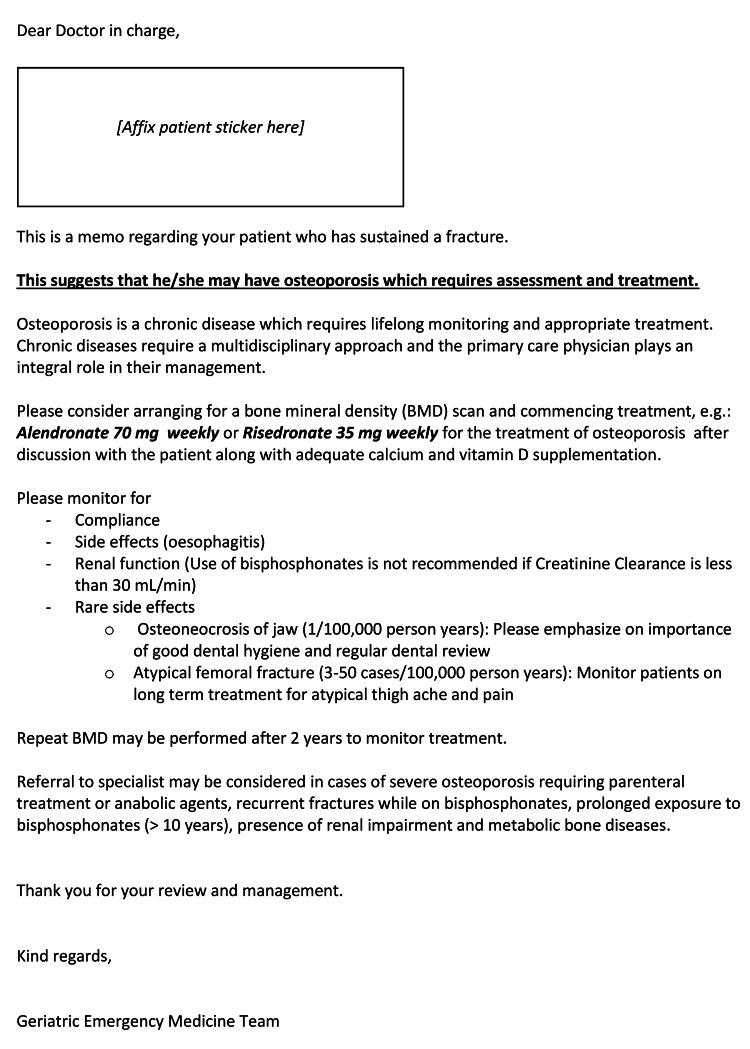
Standardized General Practitioner Memo.
